# 792 Evaluating the Efficacy of Postoperative IV Antibiotics on Graft Take: A Pilot Study

**DOI:** 10.1093/jbcr/irae036.333

**Published:** 2024-04-17

**Authors:** Seham Z Azzam, Sai Pranathi Bingi, Caezaan Keshvani,, Junior Clark, Alan Pang, John A Griswold

**Affiliations:** Texas Tech University Health Sciences Center School of Medicine, Amarillo, TX; Texas Tech University Health Sciences Center School of Medicine, Lubbock, TX; TTUHSC, Lubbock, TX; Texas Tech School of Medicine, Lubbock, TX; Texas Tech University Health Sciences Center, Lubbock, TX; Texas Tech University Health Sciences Center School of Medicine, Amarillo, TX; Texas Tech University Health Sciences Center School of Medicine, Lubbock, TX; TTUHSC, Lubbock, TX; Texas Tech School of Medicine, Lubbock, TX; Texas Tech University Health Sciences Center, Lubbock, TX; Texas Tech University Health Sciences Center School of Medicine, Amarillo, TX; Texas Tech University Health Sciences Center School of Medicine, Lubbock, TX; TTUHSC, Lubbock, TX; Texas Tech School of Medicine, Lubbock, TX; Texas Tech University Health Sciences Center, Lubbock, TX; Texas Tech University Health Sciences Center School of Medicine, Amarillo, TX; Texas Tech University Health Sciences Center School of Medicine, Lubbock, TX; TTUHSC, Lubbock, TX; Texas Tech School of Medicine, Lubbock, TX; Texas Tech University Health Sciences Center, Lubbock, TX; Texas Tech University Health Sciences Center School of Medicine, Amarillo, TX; Texas Tech University Health Sciences Center School of Medicine, Lubbock, TX; TTUHSC, Lubbock, TX; Texas Tech School of Medicine, Lubbock, TX; Texas Tech University Health Sciences Center, Lubbock, TX; Texas Tech University Health Sciences Center School of Medicine, Amarillo, TX; Texas Tech University Health Sciences Center School of Medicine, Lubbock, TX; TTUHSC, Lubbock, TX; Texas Tech School of Medicine, Lubbock, TX; Texas Tech University Health Sciences Center, Lubbock, TX

## Abstract

**Introduction:**

Burn wounds present as one of the most devastating forms of trauma. In the United States, an estimated 486,000 patients are treated for burn injuries yearly, with 40,000 requiring hospital admission. While severe burn wound survival rates have improved significantly in recent decades due to advances in the treatment of burn-related complications, approximately 5,000 patients in the U.S. die from severe burn wounds each year.

While there are many different treatments for burn wounds depending on their severity, skin grafting remains one of the most prominent and effective solutions to achieving a good patient outcome for partial and full thickness burns. One obstacle that can prevent proper healing with a skin graft is infection of the grafted wound. To mitigate this risk, IV antibiotics are given peri-operationally and are shown to increase graft survival and decrease partial graft loss suggesting potential benefit for the patient. However, there is currently little research on the efficacy of administering antibiotics postoperatively between the initial grafting procedure and undressing of the grafted wound. To better understand this association, our pilot study investigated the impact that systemic IV antibiotics have on graft outcomes when administered between graft take and dressing takedown.

**Methods:**

We queried the electronic health records of 166 burn patients who received grafting between January 1, 2011, through September 1, 2021 at a burn intensive care unit. All patients selected for this study were between 18 and 89 years of age and were divided based on those with graft success versus graft rejection. The patients were then subsequently divided into those who received post-operative antibiotics and those who did not. Using Fisher’s exact test, we compared the graft take percentages between the two groups.

**Results:**

Our study revealed no significant correlation (p=0.6054) between graft take and the use of postoperative intravenous (IV) antibiotics.

**Conclusions:**

This observation suggests that IV antibiotics given post-operative do not improve graft take in burn patients. This supports the idea that post-operative antibiotics may currently be overprescribed for patients receiving burn grafts.

**Applicability of Research to Practice:**

Although existing grafting guidelines do not discuss post-operative IV antibiotic administration, our results suggest a need for consideration. Overuse of antibiotics is well-documented to contribute to the development of antibiotic-resistant bacteria. Furthermore, burn patients are already at risk of infections due to burn injuries resulting in a state of immunosuppression that predisposes patients to infection by various pathogens.

